# Retraction: Endogenous Urotensin II Selectively Modulates Erectile Function through eNOS

**DOI:** 10.1371/journal.pone.0222415

**Published:** 2019-09-06

**Authors:** The PLOS ONE Editors

Following publication of this article [1], concerns were raised regarding similarities of western blot bands within Fig 3A. Specifically, in the p-eNOS panel, lanes 1, 2, and 5 appear similar; in the eNOS panel, lanes 1 and 5 appear similar, and lanes 3 and 4 appear similar; and in the β-actin panel, lanes 1 and 5 appear similar.

The authors indicated that the study commenced in 2009 and the original blot images underlying Fig 3 are no longer available. The authors have cooperated with the editorial follow up by providing the underlying data for the charts in Fig 3 and the blot image underlying Fig 4; the data underlying Figs 1 and 2 are also available. The corresponding author stands by the results reported in the article.

Editorial evaluation noted similarities in both band shape and in the presence of specific imperfections when the bands listed above and their surrounding backgrounds were examined closely. As the original blot images are not available, it has not been possible to resolve the concerns. In light of concerns about the preparation of Fig 3A and the reliability of the reported results, the *PLOS ONE* Editors retract this article.

RddVB, EM, FF, GC, RS did not agree with retraction. PG, EN, CI, VM did not respond. ED could not be reached.
